# Supplementary material for: The COMPASS Complex Regulates Fungal Development and Virulence through Histone Crosstalk in the Fungal Pathogen Cryptococcus neoformans
Source: J Fungi (Basel). 2023 Jun 14;9(6):672. doi: 10.3390/jof9060672 (PMC10301970; doi:10.3390/jof9060672)
Supplement: Supplementary file 1 [file jof-09-00672-s001.zip › Supplementary Table S1.pdf]

Supplementary Table S1. Resources used in this study.

Strain name	Genotype	Background	Sources and comments
H99α	WT	H99α	(30)
RL220	*set1(CNAG_01243)::NAT*	H99α	Madhani's deletion set
RL221	*bre2(CNAG_07311)::NAT*	H99α	Madhani's deletion set
RL222	*spp101(CNAG_03406)::NAT*	H99α	Madhani's deletion set
RL223	*swd1(CNAG_01828)::NAT*	H99α	Madhani's deletion set
RL224	*swd2(CNAG_03070)::NAT*	H99α	Madhani's deletion set
FZ1	*swd3(CNAG_01013)::NAT*	H99α	This study
YZ3	*pas3(CNAG_06200)::NAT*	H99α	(31)
XC141	*set1::NAT*, P_{CTR4}-*mNeonGreen-SET1-NEO*	H99α	This study
XC147	*bre2::NAT*, P_{CTR4}-*mNeonGreen-BRE2-NEO*	H99α	This study
XC148	*spp101::NAT*, P_{CTR4}-*mNeonGreen-SPP101-NEO*	H99α	This study
RL280	*swd1::NAT*, P_{SWD1}-*SWD1-mNeonGreen-NEO*	H99α	This study
RL275	*swd2::NAT*, P_{CTR4}-*mNeonGreen-SWD2-NEO*	H99α	This study
RL278	*swd3::NAT*, P_{CTR4}-*mNeonGreen-SWD3-NEO*	H99α	This study
XL280α	WT	XL280α	(35)
RL48	*set1(CND03610)::NAT*	XL280α	This study
RL54	*bre2(CNA01030)::NAT*	XL280α	This study
RL63	*spp101(CNG01770)::NAT*	XL280α	This study
RL115	*swd1(CNC03150)::NAT*	XL280α	This study
RL191	*swd2(CNC00180)::NAT*	XL280α	This study
RL83	*swd3(CND01430)::NAT*	XL280α	This study
RL97	*set1::NAT*, P_{CTR4}-*mNeonGreen-SET1-NEO*	XL280α	This study
RL118	*bre2::NAT*, P_{CTR4}-*mNeonGreen-BRE2-NEO*	XL280α	This study
RL124	*spp101::NAT*, P_{CTR4}-*mNeonGreen-SPP101-NEO*	XL280α	This study

RL170	*swd1::NAT,* P$_{SWD1}$*-SWD1-mNeonGreen-NEO*	XL280α	This study
RL208	*swd2::NAT,* P$_{CTR4}$*-mNeonGreen-SWD2-NEO*	XL280α	This study
RL127	*swd3::NAT,* P$_{CTR4}$*-mNeonGreen-SWD3-NEO*	XL280α	This study
RL105	*set1::NAT,* P$_{CTR4}$*-3×FLAG-SET1-NEO*	XL280α	This study
RL147	*bre2::NAT,* P$_{CTR4}$*-3×FLAG-BRE2-NEO*	XL280α	This study
RL143	*spp101::NAT,* P$_{CTR4}$*-3×FLAG-SPP101-NEO*	XL280α	This study
RL176	*swd1::NAT,* P$_{SWD1}$*-SWD1-4×FLAG-HYG*	XL280α	This study
RL211	*swd2::NAT,* P$_{CTR4}$*-3×FLAG-SWD2-NEO*	XL280α	This study
RL129	*swd3::NAT,* P$_{CTR4}$*-3×FLAG-SWD3-NEO*	XL280α	This study
XC27	*rtf1(CNF03680)::NAT*	XL280α	This study
XC19	*rad6(CNA08010)::NAT*	XL280α	This study
Z65	*Bre1(CNI02470)::NAT*	XL280α	(31)
XC109	*rtf1::NAT,* P$_{CTR4}$*-3xFLAG-RTF1-NEO*	XL280α	This study
XC64	*rad6::NAT,* P$_{RAD6}$*-RAD6-HYG-GFP*	XL280α	This study
YJ9	*bre1::NAT,* P$_{CTR4}$*-3xFLAG-BRE1-NEO*	XL280α	This study
XL280 **a**	WT	XL280**a**	(37)
RL182	*set1::NAT*	XL280**a**	This study
RL184	*bre2::NAT*	XL280**a**	This study
RL185	*spp101::NAT*	XL280**a**	This study
RL186	*swd1::NAT*	XL280**a**	This study
RL217	*swd2::NAT*	XL280**a**	This study
RL189	*swd3::NAT*	XL280**a**	This study
YJ27	*CNA06650::NAT*	XL280α	This study
YJ31	*CNA01460::NAT*	XL280α	This study
XC121	*CNG01680::NAT*	XL280α	This study
XC125	*CNA06060::NAT*	XL280α	This study
XC126	*CNN01130::NAT*	XL280α	This study
TW36	*CNI02100::NAT*	XL280α	This study
TW33	*CND06330::NAT*	XL280α	This study
TW30	*CNB05630::NAT*	XL280α	This study
RL195	*CND02180::NAT*	XL280α	This study
RL199	*CNC01350::NAT*	XL280α	This study
RL203	*CNC02620::NAT*	XL280α	This study

TC6	*CNI03780::NAT*	XL280α	This study
TC8	*CNE02660::NAT*	XL280α	This study
TC5	*CNI03230::NAT*	XL280α	This study
RL266	*CNC02870::NAT*	XL280α	This study
RL237	*CNAG_02006::NAT*	H99α	Madhani's deletion set
RL238	*CNAG_03396::NAT*	H99α	Madhani's deletion set
RL239	*CNAG_03360::NAT*	H99α	Madhani's deletion set
RL240	*CNAG_03528::NAT*	H99α	Madhani's deletion set
RL241	*CNAG_03886::NAT*	H99α	Madhani's deletion set
RL242	*CNAG_00718::NAT*	H99α	Madhani's deletion set
RL243	*CNAG_04313::NAT*	H99α	Madhani's deletion set
RL244	*CNAG_05613::NAT*	H99α	Madhani's deletion set
RL245	*CNAG_06548::NAT*	H99α	Madhani's deletion set
RL246	*CNAG_06356::NAT*	H99α	Madhani's deletion set
RL247	*CNAG_07667::NAT*	H99α	Madhani's deletion set
RL248	*CNAG_03592::NAT*	H99α	Madhani's deletion set
RL249	*CNAG_05386::NAT*	H99α	Madhani's deletion set
RL250	*CNAG_07464::NAT*	H99α	Madhani's deletion set
RL251	*CNAG_02366::NAT*	H99α	Madhani's deletion set
RL252	*CNAG_02415::NAT*	H99α	Madhani's deletion set
RL253	*CNAG_02088::NAT*	H99α	Madhani's deletion set
RL254	*CNAG_04639::NAT*	H99α	Madhani's deletion set
RL255	*CNAG_05768::NAT*	H99α	Madhani's deletion set
RL256	*CNAG_01824::NAT*	H99α	Madhani's deletion set

RL257	*CNAG_02240::NAT*	H99α	Madhani's deletion set
RL258	*CNAG_05195::NAT*	H99α	Madhani's deletion set
RL259	*CNAG_00556::NAT*	H99α	Madhani's deletion set
RL260	*CNAG_00020::NAT*	H99α	Madhani's deletion set
RL261	*CNAG_00876::NAT*	H99α	Madhani's deletion set
RL262	*CNAG_00690::NAT*	H99α	Madhani's deletion set

**Plasmid**	**Genotype**	**Background**	**Sources and comments**
pYZ99	*1034-Sun-HYG-4xFLAG*	1034-Sun	(48)
pYZ100	*1032-Sun-HYG-GFP*	1032-Sun	(48)
pYZ090	*1038-Sun-HYG-mCherry*	1038-Sun	(48)
pYZ170	P_{CTR4}-*mNeonGreen-NEO*	pFZ1	This study
pYZ175	P_{CTR4}-*3×FLAG-NEO*	pFZ3	This study
pYZ25	P_{TEF1}-*mNeonGreen-NEO*	pUC19	This study
pYZ216	P_{CTR4}-*mNeonGreen-SET1-NEO*	pFZ1	This study
pYZ230	P_{CTR4}-*mNeonGreen-BRE2-NEO*	pFZ1	This study
pYZ264	P_{CTR4}-*mNeonGreen-SPP101-NEO*	pFZ1	This study
pYZ325	P_{SWD1}-*SWD1-mNeonGreen-NEO*	pUC19	This study
pYZ349	P_{CTR4}-*mNeonGreen-SWD2-NEO*	pFZ1	This study
pYZ241	P_{CTR4}-*mNeonGreen-SWD3-NEO*	pFZ1	This study
pYZ218	P_{CTR4}-*3×FLAG-SET1-NEO*	pFZ3	This study
pYZ233	P_{CTR4}-*3×FLAG-BRE2-NEO*	pFZ3	This study
pYZ267	P_{CTR4}-*3×FLAG-SPP101-NEO*	pFZ3	This study
pYZ340	P_{SWD1}-*SWD1-4×FLAG-HYG*	1034-Sun	This study
pYZ352	P_{CTR4}-*3×FLAG-SWD2-NEO*	pFZ3	This study
pYZ243	P_{CTR4}-*3×FLAG-SWD3-NEO*	pFZ3	This study
pYZ281	P_{CTR4}-*3xFLAG-RTF1-NEO*	pFZ3	This study
pYZ188	P_{RAD6}-*RAD6-HYG-GFP*	1032-Sun	This study
pYZ262	P_{CTR4}-*3xFLAG-BRE1-NEO*	pFZ3	This study

Primer name	Sequence (5' to 3')	Description
M13F	GTAAAACGACGGCCAGT	NAT/NEO/HYG cassette and TRACE constructs from plasmid
M13R	CAGGAAACAGCTATGAC	NAT/NEO/HYG cassette and TRACE constructs from plasmid
ZhaoLab0003/YZ	TTGGATGCTGGATGCTGGGT	NAT-F
ZhaoLab0004/YZ	CCGTCTTCACCTGCATCTGATT	NAT-split-R
ZhaoLab0015/YZ	TGTGGATGCTGGCGGAGGATA	NAT-split-R for positive PCR of mutant
ZhaoLab0192/YZ	AACTGAGATACCTACAGCGTGAG	gRNA-scaffold-Far-R
ZhaoLab0193/YZ	ACTCCCTGGTCCCATCCCT	CnU6-Far-F
ZhaoLab0011/YZ	CCATCGATTTGCATTAGAACTAAAAA CAAAGCA	U6 promoter NF
ZhaoLab0012/YZ	CCGCTCGAGTAAAACAAAAAAGCAC CGAC	gRNA nested NR
ZhaoLab0054/YZ	GATAGATACTGAGGAGGACAT	PGPD1_F for Cas9
ZhaoLab0055/YZ	GGGCCCCTCTTCACGTGG	TGPD1_R for Cas9
ZhaoLab0013/YZ	AGACTCCACAGCCTAAGATCAACAG TATACCCTGCCGGTG	*SH2* gRNA, paired with 0193 (U6 promoter FLF)
ZhaoLab0014/YZ	GATCTTAGGCTGTGGAGTCTGTTTTA GAGCTAGAAATAGCAAGTT	*SH2* gRNA, paired with 0192 (gRNA terminator FR)
ZhaoLab0105/YZ	GTTCTCTGACCCAAAACATCGTTTTA GAGCTAGAAATAGCAAGTT	*SH3* gRNA, paired with 0193 (U6 promoter FLF)
ZhaoLab0106/YZ	GATGTTTTGGGTCAGAGAACAACAG TATACCCTGCCGGTG	*SH3* gRNA, paired with 0192 (gRNA terminator FR)
ZhaoLab0188/YZ	CGAAGGATGGTTGTCGCTC	screening insertion into SH3 in serotype D
ZhaoLab0189/YZ	GTATCGTCTTGCTCTTCATTCC	screening insertion into SH3 in serotype D
ZhaoLab0190/YZ	GTTGTTTCAGGCCTGCGGATG	screening insertion into SH2 in serotype A
ZhaoLab0191/YZ	GACTCATTCCTATGCCGTTC	screening insertion into SH2 in serotype A
ZhaoLab0553/FZ	GGAAGATATGGTGTGGGA	*SWD3* deletion in H99 LF
ZhaoLab0554/FZ	CTGGCCGTCGTTTTACGGCCAATAC CTACGACTGC	*SWD3* deletion in H99 LR
ZhaoLab0555/FZ	TGAATGCTCCAGGAATAGA	*SWD3* deletion in H99 NLF

ZhaoLab0556/FZ	GTCATAGCTGTTTCCTGAAGCCTGCA TTGTTGGGG	*SWD3* deletion in H99 RF
ZhaoLab0557/FZ	CGATTATACAGCGGTGATGC	*SWD3* deletion in H99 RR
ZhaoLab0558/FZ	GATGAACTTCTGATGCTCC	*SWD3* deletion in H99 NRR
ZhaoLab0559/FZ	ATAATATGGGACGTCAGAAGGTTTTA GAGCTAGAAATAGCAAGTT	*SWD3* deletion in H99 gRNA, paired with 0192 (gRNA terminator FR)
ZhaoLab0560/FZ	CTTCTGACGTCCCATATTATAACAGT ATACCCTGCCGGTG	*SWD3* deletion in H99 gRNA, paired with 0193 (U6 promoter FLF)
ZhaoLab0561/FZ	ATAGTTCCAGTGCCTCCT	*SWD3* deletion in H99 Test F
ZhaoLab0562/FZ	GTAAGTACCATTTCACCCTC	*SWD3* deletion in H99 Test R
ZhaoLab0347/YJ	TGAGCCAATGTAGATACG	*CNA06650* deletion LF
ZhaoLab0348/YJ	CTGGCCGTCGTTTTAC ACGGAGTGATCTGAAGAG	*CNA06650* deletion LR
ZhaoLab0349/YJ	TCAAGCCCGACGACTCAG	*CNA06650* deletion NLF
ZhaoLab0350/YJ	GTCATAGCTGTTTCCTG GCCTCCTTCTTGAGTTCTTT	*CNA06650* deletion RF
ZhaoLab0351/YJ	GCGAGTCTTCGTCCTGTG	*CNA06650* deletion RR
ZhaoLab0352/YJ	TCTTTCTTTGCGGCGTGT	*CNA06650* deletion NRR
ZhaoLab0353/YJ	ACGTTGGTTTCGCTTGGTGT GTTTTAGAGCTAGAAATAGCAAGTT	*CNA06650* deletion gRNA, paired with 0192 (gRNA terminator FR)
ZhaoLab0354/YJ	ACACCAAGCGAAACCAACGT AACAGTATACCCTGCCGGTG	*CNA06650* deletion gRNA, paired with 0193 (U6 promoter FLF)
ZhaoLab0355/YJ	CAAGATAACGGAGAAGTGG	*CNA01460* deletion LF
ZhaoLab0356/YJ	CTGGCCGTCGTTTTAC TTTGTAAAGCTATGCTAG	*CNA01460* deletion LR
ZhaoLab0357/YJ	ATAACGGAGAAGTGGACG	*CNA01460* deletion NLF
ZhaoLab0358/YJ	GTCATAGCTGTTTCCTG CGTGGCCCATGCCACATG	*CNA01460* deletion RF
ZhaoLab0359/YJ	GTTGGACCAAAGCGAGAA	*CNA01460* deletion RR
ZhaoLab0360/YJ	TGGTCACCCAGTGTTCCT	*CNA01460* deletion NRR
ZhaoLab0361/YJ	GATCAAGGGTACTAGGCCTA GTTTTAGAGCTAGAAATAGCAAGTT	*CNA01460* deletion gRNA, paired with 0192 (gRNA terminator FR)
ZhaoLab0362/YJ	TAGGCCTAGTACCCTTGATC AACAGTATACCCTGCCGGTG	*CNA01460* deletion gRNA, paired with 0193 (U6 promoter FLF)
ZhaoLab0371/XC	CCTACCTGCCGCATTTGT	*CNG01680* deletion LF
ZhaoLab0372/XC	CTGGCCGTCGTTTTAC CCCTGCTGGAGTTCTTCG	*CNG01680* deletion LR

ZhaoLab0373/XC	CGCATTTGTGCTTGTTGA	*CNG01680* deletion NLF
ZhaoLab0374/XC	GTCATAGCTGTTTCCTG CTCATACAACAAATGTGG	*CNG01680* deletion RF
ZhaoLab0375/XC	CATTGATGTCTTGGCAGA	*CNG01680* deletion RR
ZhaoLab0376/XC	ATCCTGCTACTGGTAACTG	*CNG01680* deletion NRR
ZhaoLab0377/XC	GACTTGCGTGCCCACGTCTGGTTTT AGAGCTAGAAATAGCAAGTT	*CNG01680* deletion gRNA, paired with 0192 (gRNA terminator FR)
ZhaoLab0378/XC	CAGACGTGGGCACGCAAGTCAACAG TATACCCTGCCGGTG	*CNG01680* deletion gRNA, paired with 0193 (U6 promoter FLF)
ZhaoLab0379/XC	TACCTCCTTTCACGGTCTC	*CNA06060*deletion LF
ZhaoLab0380/XC	CTGGCCGTCGTTTTACAGCTGATGT GTTGTTTAT	*CNA06060*deletion LR
ZhaoLab0381/XC	CTTTCACGGTCTCATTGTC	*CNA06060*deletion NLF
ZhaoLab0382/XC	GTCATAGCTGTTTCCTGGAATTAGGC TTGTACGAA	*CNA06060*deletion RF
ZhaoLab0383/XC	TATAGTGGCAAAGGGAGT	*CNA06060*deletion RR
ZhaoLab0384/XC	TGGGGTTGGTAATCCGTT	*CNA06060*deletion NRR
ZhaoLab0385/XC	GTCGTCAGAGGCCTTGTTGGGTTTT AGAGCTAGAAATAGCAAGTT	*CNA06060*deletion gRNA, paired with 0192 (gRNA terminator FR)
ZhaoLab0386/XC	CCAACAAGGCCTCTGACGACAACAG TATACCCTGCCGGTG	*CNA06060*deletion gRNA, paired with 0193 (U6 promoter FLF)
ZhaoLab0387/XC	ATGTTGGTGACAGATAAGG	*CNN01130* deletion LF
ZhaoLab0388/XC	CTGGCCGTCGTTTTACAGTGACACT GGATAGTTT	*CNN01130* deletion LR
ZhaoLab0389/XC	TTACAGTCAGTAGCGTAAA	*CNN01130* deletion NLF
ZhaoLab0390/XC	GTCATAGCTGTTTCCTG AGGACCTCCGAGGAAATT	*CNN01130* deletion RF
ZhaoLab0391/XC	CCTGGCTGAGTACAGTAGTTG	*CNN01130* deletion RR
ZhaoLab0392/XC	GTTGTAGATGGCGGTATA	*CNN01130* deletion NRR
ZhaoLab0393/XC	GCTTTGAAGGAGTTTGATGGGTTTTA GAGCTAGAAATAGCAAGTT	*CNN01130* deletion gRNA, paired with 0192 (gRNA terminator FR)
ZhaoLab0394/XC	CCATCAAACTCCTTCAAAGCCAACAG TATACCCTGCCGGTG	*CNN01130* deletion gRNA, paired with 0193 (U6 promoter FLF)
ZhaoLab0395/TW	TAAGGAGATGCTACCCTG	*CNI02100*deletion LF
ZhaoLab0396/TW	CTGGCCGTCGTTTTAC TGATAAGATATAAAAAAG	*CNI02100*deletion LR
ZhaoLab0397/TW	AGGAGGATGTCCCCAGTT	*CNI02100*deletion NLF
ZhaoLab0398/TW	GTCATAGCTGTTTCCTG	*CNI02100*deletion RF
ZhaoLab0399/TW	TTGGTCTTAGGCTTTGTT	*CNI02100*deletion RR

ZhaoLab0400/TW	TTACTTGGGCGAAACAGC	*CNI02100*deletion NRR
ZhaoLab0401/TW	AGGGGCGACGGAGTTCGTTA GTTTTAGAGCTAGAAATAGCAAGTT	*CNI02100*deletion gRNA, paired with 0192 (gRNA terminator FR)
ZhaoLab0402/TW	TAACGAACTCCGTCGCCCCT AACAGTATACCCTGCCGGTG	*CNI02100*deletion gRNA, paired with 0193 (U6 promoter FLF)
ZhaoLab0403/TW	AAAAGGATGGAAGAGCAA	*CND06330* deletion LF
ZhaoLab0404/TW	CTGGCCGTCGTTTTAC TCATGCATCCCTAATTGC	*CND06330* deletion LR
ZhaoLab0405/TW	GAGTTGTACCGTTGTTGG	*CND06330* deletion NLF
ZhaoLab0406/TW	GTCATAGCTGTTTCCTG AGCCGGTGGATTGGTTAG	*CND06330* deletion RF
ZhaoLab0407/TW	GTGGCAAGATCGTGATAG	*CND06330* deletion RR
ZhaoLab0408/TW	GTCGTTTAAGGACAGAAG	*CND06330* deletion NRR
ZhaoLab0409/TW	GATCTGGATCGGGAAGGCCG GTTTTAGAGCTAGAAATAGCAAGTT	*CND06330* deletion gRNA, paired with 0192 (gRNA terminator FR)
ZhaoLab0410/TW	CGGCCTTCCCGATCCAGATC AACAGTATACCCTGCCGGTG	*CND06330* deletion gRNA, paired with 0193 (U6 promoter FLF)
ZhaoLab0411/TW	AAGGAAGACTACGATGCT	*CNB05630* deletion LF
ZhaoLab0412/TW	CTGGCCGTCGTTTTAC ATGTGAAAACTCATTTGG	*CNB05630* deletion LR
ZhaoLab0413/TW	CAATTTCCCCAAATGGCT	*CNB05630* deletion NLF
ZhaoLab0414/TW	GTCATAGCTGTTTCCTG CGGGGTTAAAGGGAAGAC	*CNB05630* deletion RF
ZhaoLab0415/TW	AGGAGCAGGAGCAGAAGC	*CNB05630* deletion RR
ZhaoLab0416/TW	TCCACAAGACTGTCGAGGTA	*CNB05630* deletion NRR
ZhaoLab0417/TW	ATTCGGCGAGGGATTTGGAG GTTTTAGAGCTAGAAATAGCAAGTT	*CNB05630* deletion gRNA, paired with 0192 (gRNA terminator FR)
ZhaoLab0418/TW	CTCCAAATCCCTCGCCGAAT AACAGTATACCCTGCCGGTG	*CNB05630* deletion gRNA, paired with 0193 (U6 promoter FLF)
ZhaoLab0419/RL	TCGGCTGCACAATGGGTT	*CND02180* deletion LF
ZhaoLab0420/RL	CTGGCCGTCGTTTTACTCGGCATCG GTATTAGGG	*CND02180* deletion LR
ZhaoLab0421/RL	TTCCTGTACCAACGCCTCA	*CND02180* deletion NLF
ZhaoLab0422/RL	GTCATAGCTGTTTCCTGTCAGTAGCA GGAGTGTATGG	*CND02180* deletion RF
ZhaoLab0423/RL	TTTAATTCTGCCTCCACAT	*CND02180* deletion RR
ZhaoLab0424/RL	CCGGCCATCGTTGTCAGT	*CND02180* deletion NRR

ZhaoLab0425/RL	GTAGTGGACCATTCACCGGA GTTTTAGAGCTAGAAATAGCAAGTT	*CND02180* deletion gRNA, paired with 0192 (gRNA terminator FR)
ZhaoLab0426/RL	TCCGGTGAATGGTCCACTAC AACAGTATACCCTGCCGGTG	*CND02180* deletion gRNA, paired with 0193 (U6 promoter FLF)
ZhaoLab0427/RL	ATTATCCGTCTTCCGTTCT	*CNC01350* deletion LF
ZhaoLab0428/RL	CTGGCCGTCGTTTTACTGTGTTTGAT CTTGCAAAAA	*CNC01350* deletion LR
ZhaoLab0429/RL	GTTCTCCTCCGTCGTCAT	*CNC01350* deletion NLF
ZhaoLab0430/RL	GTCATAGCTGTTTCCTGACAGGTGTT CGACCGTATGG	*CNC01350* deletion RF
ZhaoLab0431/RL	ATTCTGGCTTCGTTCCCT	*CNC01350* deletion RR
ZhaoLab0432/RL	TCTGTCGCTCAGGCTCAT	*CNC01350* deletion NRR
ZhaoLab0433/RL	GTGGTGTCGCAAAACAGTGG GTTTTAGAGCTAGAAATAGCAAGTT	*CNC01350* deletion gRNA, paired with 0192 (gRNA terminator FR)
ZhaoLab0434/RL	CCACTGTTTTGCGACACCAC AACAGTATACCCTGCCGGTG	*CNC01350* deletion gRNA, paired with 0193 (U6 promoter FLF)
ZhaoLab0435/RL	AATGTTTCTGAATGCAAG	*CNC02620* deletion LF
ZhaoLab0436/RL	CTGGCCGTCGTTTTACAGGGTCATG GTCGTTATA	*CNC02620* deletion LR
ZhaoLab0437/RL	TTGTATTTGCTGTCGCTA	*CNC02620* deletion NLF
ZhaoLab0438/RL	GTCATAGCTGTTTCCTGTGTGATGAT GGAAGCCAAAG	*CNC02620* deletion RF
ZhaoLab0439/RL	TCGGAGGACATCGAAGGTA	*CNC02620* deletion RR
ZhaoLab0440/RL	CCTGGAGGACGGACATTG	*CNC02620* deletion NRR
ZhaoLab0441/RL	GTTTGGTTTGGAACTGGTGC GTTTTAGAGCTAGAAATAGCAAGTT	*CNC02620* deletion gRNA, paired with 0192 (gRNA terminator FR)
ZhaoLab0442/RL	GCACCAGTTCCAAACCAAAC AACAGTATACCCTGCCGGTG	*CNC02620* deletion gRNA, paired with 0193 (U6 promoter FLF)
ZhaoLab0510/RL	CGCAATCACATTCGACCTT	*CNI03780* deletion LF
ZhaoLab0511/RL	CTGGCCGTCGTTTTACGTTGGAATTT GGATACCTGT	*CNI03780* deletion LR
ZhaoLab0512/RL	GGGGAGTGTAGCTTTGAGT	*CNI03780* deletion NLF
ZhaoLab0513/RL	GTCATAGCTGTTTCCTGCGTAAGTCA AAAATACCTTT	*CNI03780* deletion RF
ZhaoLab0514/RL	AGACAGCCGACAGAGCAG	*CNI03780* deletion RR
ZhaoLab0515/RL	TGCGCTGGGTTGGTGATG	*CNI03780* deletion NRR
ZhaoLab0516/RL	ACAGAATTTGGTCTCGTCTT GTTTTAGAGCTAGAAATAGCAAGTT	*CNI03780* deletion gRNA, paired with 0192 (gRNA terminator FR)

ZhaoLab0517/RL	AAGACGAGACCAAATTCTGT AACAGTATACCCTGCCGGTG	*CNI03780* deletion gRNA, paired with 0193 (U6 promoter FLF)
ZhaoLab0518/RL	TATTTATTTCTCGCTTCG	*CNI03780* deletion Test F
ZhaoLab0519/RL	TCAAATGATACTGCTGTGA	*CNI03780* deletion Test R
ZhaoLab0520/RL	TGTCTGGCGGGTGGCTTCT	*CNE02660* deletion LF
ZhaoLab0521/RL	CTGGCCGTCGTTTTACGGGAGGGCG ACTTCCCTGT	*CNE02660* deletion LR
ZhaoLab0522/RL	TTGCGAGGTTGAGATTGC	*CNE02660* deletion NLF
ZhaoLab0523/RL	GTCATAGCTGTTTCCTGTTTCATATT AGATTGTATGA	*CNE02660* deletion RF
ZhaoLab0524/RL	GGTCAGTAGCTTTTCTTG	*CNE02660* deletion RR
ZhaoLab0525/RL	TATCTCCTGACGAAGTCC	*CNE02660* deletion NRR
ZhaoLab0526/RL	AAAACGCAAGCACGGGCGAA	*CNE02660* deletion gRNA, paired with 0192 (gRNA terminator FR)
ZhaoLab0527/RL	TTCGCCCGTGCTTGCGTTTT AACAGTATACCCTGCCGGTG	*CNE02660* deletion gRNA, paired with 0193 (U6 promoter FLF)
ZhaoLab0528/RL	AGGCTGTTCGTACATATCTCA	*CNE02660* deletion Test F
ZhaoLab0529/RL	GCTATCGCTGGCTTCTTC	*CNE02660* deletion Test R
ZhaoLab0530/RL	AAGCGAATGCCACTTGGG	*CNI03230* deletion LF
ZhaoLab0531/RL	CTGGCCGTCGTTTTACGGCGGATGA GATTGGGCTA	*CNI03230* deletion LR
ZhaoLab0532/RL	TTTGCTGGTGACACTGCG	*CNI03230* deletion NLF
ZhaoLab0533/RL	GTCATAGCTGTTTCCTGAGGGGCGC TGAAGGCTTT	*CNI03230* deletion RF
ZhaoLab0534/RL	ATGGCATTAGAAGAGGACATAC	*CNI03230* deletion RR
ZhaoLab0535/RL	CCCTCTTTCCCCTCCTAT	*CNI03230* deletion NRR
ZhaoLab0536/RL	AAATGGTACGGTGTTAATAC GTTTTAGAGCTAGAAATAGCAAGTT	*CNI03230* deletion gRNA, paired with 0192 (gRNA terminator FR)
ZhaoLab0537/RL	GTATTAACACCGTACCATTT AACAGTATACCCTGCCGGTG	*CNI03230* deletion gRNA, paired with 0193 (U6 promoter FLF)
ZhaoLab0538/RL	CCTACTCTTCGGTTCAAT	*CNI03230* deletion Test F
ZhaoLab0539/RL	ACAAGCACAGTCAAAGCC	*CNI03230* deletion Test R
ZhaoLab0609/RL	TAGAGGGTCTCCATCGTTT	*CNC02870* deletion LF
ZhaoLab0610/RL	CTGGCCGTCGTTTTACTGTTATAGTT AGAGTTTGTA	*CNC02870* deletion LR
ZhaoLab0611/RL	AGAAGCGACAGGAGTTGG	*CNC02870* deletion NLF
ZhaoLab0612/RL	GTCATAGCTGTTTCCTGACGTAAAGC TATTCCAGTTT	*CNC02870* deletion RF

ZhaoLab0613/RL	CTTCCTGTTCTTGCCTCT	*CNC02870* deletion RR
ZhaoLab0614/RL	CTGGGATGGATGAGGATGTT	*CNC02870* deletion NRR
ZhaoLab0615/RL	AGAGGGCTTGGGGACCTCTT GTTTTAGAGCTAGAAATAGCAAGTT	*CNC02870* deletion gRNA, paired with 0192 (gRNA terminator FR)
ZhaoLab0616/RL	AAGAGGTCCCCAAGCCCTCT AACAGTATACCCTGCCGGTG	*CNC02870* deletion gRNA, paired with 0193 (U6 promoter FLF)
ZhaoLab0617/RL	AAGTAGCTGCACCCAACA	*CNC02870* deletion Test F
ZhaoLab0618/RL	TCACCTTGACTGCCGATT	*CNC02870* deletion Test R
ZhaoLab0028/YZ	TGACGGAACCGAACAGAC	*SET1* deletion in XL280 LF
ZhaoLab0029/YZ	CTGGCCGTCGTTTTACTTGTGACTCG GAGCAGCT	*SET1* deletion in XL280 LR
ZhaoLab0030/YZ	TCACCCAAATCCTGTAAG	*SET1* deletion in XL280 NLF
ZhaoLab0031/YZ	GTCATAGCTGTTTCCTGGCTGACGG GCGTCGATACTTT	*SET1* deletion in XL280 RF
ZhaoLab0032/YZ	AGGAGGCAGGGAGATGGTGG	*SET1* deletion in XL280 RR
ZhaoLab0033/YZ	TGTGGGAGTGAAGAGGGA	*SET1* deletion in XL280 NRR
ZhaoLab0034/YZ	AGAGTGGTGTTGGACGGCAG GTTTTAGAGCTAGAAATAGCAAGTT	*SET1* deletion in XL280 gRNA, paired with 0192 (gRNA terminator FR)
ZhaoLab0035/YZ	CTGCCGTCCAACACCACTCT AACAGTATACCCTGCCGGTG	*SET1* deletion in XL280 gRNA, paired with 0193 (U6 promoter FLF)
ZhaoLab0287/YZ	GATTTGTATTCCGTTACTCCC	*SET1* deletion in XL280 Test F
ZhaoLab0288/YZ	GCCGTCCAACACCACTCT	*SET1* deletion in XL280 Test R
ZhaoLab0087/YZ	ATAAGGCCGGCCGCACCTCATGAGA AAGGTG	*SET1* forward with FseI for mNeonGreen tag
ZhaoLab0088/YZ	ATATTAATTAATCAGTTGAGCCAGCC TCG	*SET1* reverse with PacI for mNeonGreen tag
ZhaoLab0107/RL	ATAAGCGGCCGCCCCAAATCCTGTA AGTAGC	*SET1* forward with NotI for FLAG tag
ZhaoLab0108/RL	ATAAGCGGCCGCGTTGAGCCAGCCT CGGCA	*SET1* reverse with NotI for FLAG tag
ZhaoLab0056/YZ	CCAAGACGGCGGAAACAT	*BRE2* deletion in XL280 LF
ZhaoLab0057/YZ	CTGGCCGTCGTTTTACGAAGGGATG GGACGGAGA	*BRE2* deletion in XL280 LR
ZhaoLab0058/YZ	CTGGCATAACCCAACAAG	*BRE2* deletion in XL280 NLF

ZhaoLab0059/YZ	GTCATAGCTGTTTCCTGGCGAAGGT ATGAAGAACG	*BRE2* deletion in XL280 RF
ZhaoLab0060/YZ	AGACTGAGAATCGAGCAAC	*BRE2* deletion in XL280 RR
ZhaoLab0061/YZ	TCGAGCAACTGTCGAATA	*BRE2* deletion in XL280 NRR
ZhaoLab0062/YZ	ATACGCGCAGGATCGGAAACGTTTT AGAGCTAGAAATAGCAAGTT	*BRE2* deletion in XL280 gRNA, paired with 0192 (gRNA terminator FR)
ZhaoLab0063/YZ	GTTTCCGATCCTGCGCGTATAACAGT ATACCCTGCCGGTG	*BRE2* deletion in XL280 gRNA, paired with 0193 (U6 promoter FLF)
ZhaoLab0283/YZ	AAAATACGCCTGGCTCTT	*BRE2* deletion in XL280 Test F
ZhaoLab0284/YZ	CACCCTCACTACTGACAATCTA	*BRE2* deletion in XL280 Test R
ZhaoLab0239/YZ	ATAAGGCCGGCCTCGTTAGAAAACA GGCTATC	*BRE2* forward with FseI
ZhaoLab0240/YZ	CCTTAATTAACTACCAGTTGATGCCT TCAA	*BRE2* reverse with PacI
ZhaoLab0072/YZ	GGGGTGGAGACAAGGGAA	*SPP101* deletion in XL280 LF
ZhaoLab0073/YZ	CTGGCCGTCGTTTTACACCGCAATC AAGCAGTTA	*SPP101* deletion in XL280 LR
ZhaoLab0074/YZ	GACGGATGTATGTAGAGGG	*SPP101* deletion in XL280 NLF
ZhaoLab0075/YZ	GTCATAGCTGTTTCCTGAAGCAGCTA GACAGTCCG	*SPP101* deletion in XL280 RF
ZhaoLab0076/YZ	ACTTTCGTGATTGGTCGT	*SPP101* deletion in XL280 RR
ZhaoLab0077/YZ	TGTAAATGTTCTCGCAAAG	*SPP101* deletion in XL280 NRR
ZhaoLab0078/YZ	ATCCTCATCCTTCACCATCGGTTTTA GAGCTAGAAATAGCAAGTT	*SPP101* deletion in XL280 gRNA, paired with 0192 (gRNA terminator FR)
ZhaoLab0079/YZ	CGATGGTGAAGGATGAGGATAACAG TATACCCTGCCGGTG	*SPP101* deletion in XL280 gRNA, paired with 0193 (U6 promoter FLF)
ZhaoLab0285/YZ	CTGTACCTGTTGGCTCATT	*SPP101* deletion in XL280 Test F
ZhaoLab0286/YZ	GTGCCTCACATCTTTATTTC	*SPP101* deletion in XL280 Test R
ZhaoLab0257/YZ	ATAAGGCCGGCCTCCGAAGCTGGAG AAAACA	*SPP101* forward with FseI
ZhaoLab0258/YZ	CCTTAATTAACTATTTGCGCTTCACA GC	*SPP101* reverse with PacI
ZhaoLab0196/YZ	TGGTAGAGTCGGACAGAG	*SWD1* deletion in XL280 LF

ZhaoLab0197/YZ	CTGGCCGTCGTTTTACAGCAGACAG ACGGAGTAG	*SWD1* deletion in XL280 LR
ZhaoLab0198/YZ	GCCGAGTATTAGTTTGTTG	*SWD1* deletion in XL280 NLF
ZhaoLab0199/YZ	GTCATAGCTGTTTCCTGCGGAACTTG AGTATGTGAG	*SWD1* deletion in XL280 RF
ZhaoLab0200/YZ	TCAATATCTACACGGAAGC	*SWD1* deletion in XL280 RR
ZhaoLab0201/YZ	TTTCAAGGAAATCTGTCG	*SWD1* deletion in XL280 NRR
ZhaoLab0194/YZ	GAAGTCAGCAGAACTTACTG GTTTTAGAGCTAGAAATAGCAAGTT	*SWD1* deletion in XL280 gRNA, paired with 0192 (gRNA terminator FR)
ZhaoLab0195/YZ	CAGTAAGTTCTGCTGACTTC AACAGTATACCCTGCCGGTG	*SWD1* deletion in XL280 gRNA, paired with 0193 (U6 promoter FLF)
ZhaoLab0289/YZ	GAGGTTGTGCCCATAAAG	*SWD1* deletion in XL280 Test F
ZhaoLab0290/YZ	AGCGGATAGAAGGTAGCG	*SWD1* deletion in XL280 Test R
ZhaoLab0333/YZ	CTCGGTACCCGGGGCTGGTAGAGTC GGACAGAGAGTCTTTCA	*SWD1* forward with NotI for mNeonGreen tag
ZhaoLab0334/YZ	TGGACACCATTGCGATCGCAGTTCC GGCGTCATCACT	*SWD1* reverse with AsiSI for mNeonGreen tag
ZhaoLab0341/YZ	CGCGCCGCATGCTGCGGCCGCAGT TCCGGCGTCATCACT	*SWD1* forward with NotI for FLAG tag
ZhaoLab0342/YZ	GATGCATGCTCGAGCTGGTAGAGTC GGACAGAGAGTCT	*SWD1* reverse with NotI for FLAG tag
ZhaoLab0459/RL	TATCATCTGGACTTGCCTAT	*SWD2* deletion in XL280 LF
ZhaoLab0460/RL	CTGGCCGTCGTTTTACGATGCAATCT ACCTTACT	*SWD2* deletion in XL280 LR
ZhaoLab0461/RL	GGACTTGCCTATAAACAGC	*SWD2* deletion in XL280 NLF
ZhaoLab0462/RL	GTCATAGCTGTTTCCTGTTTCAATTG TTACTTCTAG	*SWD2* deletion in XL280 RF
ZhaoLab0463/RL	GCTGAACGGTCACTTTAT	*SWD2* deletion in XL280 RR
ZhaoLab0464/RL	CTTTATAGCTCAAAAGGA	*SWD2* deletion in XL280 NRR
ZhaoLab0465/RL	GATGGGACAAAGAAGCTGAG GTTTTAGAGCTAGAAATAGCAAGTT	*SWD2* deletion gRNA in XL280, paired with 0192 (gRNA terminator FR)
ZhaoLab0466/RL	CTCAGCTTCTTTGTCCCATC AACAGTATACCCTGCCGGTG	*SWD2* deletion gRNA in XL280, paired with 0193 (U6 promoter FLF)
ZhaoLab0467/RL	GCCTCCAACATCATTCAC	*SWD2* deletion in XL280 Test F

ZhaoLab0468/RL	TCATCGGGTCTCAGTTTAT	*SWD2* deletion in XL280 Test R
ZhaoLab0487/YZ	GTGGCTCTGGGCCGGCCATGGATAC TCCTGGCGCC	*SWD2* forward with FseI for mNeonGreen tag
ZhaoLab0488/YZ	CTGCTACTGTAACCCTTAATCTACCA CCCCTCGGCGGC	*SWD2* reverse with PacI
ZhaoLab0489/YZ	GTGGCGGTGGGCCGGCCATGGATA CTCCTGGCGCC	*SWD2* forward with FseI for FLAG tag
ZhaoLab0204/YZ	CCGTCATTCGTGCTTACT	*SWD3* deletion in XL280 LF
ZhaoLab0205/YZ	CTGGCCGTCGTTTTACATTGCTGGC GCAGTGTAT	*SWD3* deletion in XL280 LR
ZhaoLab0206/YZ	GGGCCTGCTGCAAAACAT	*SWD3* deletion in XL280 NLF
ZhaoLab0207/YZ	GTCATAGCTGTTTCCTGTATGTTGGG CTGATTTGG	*SWD3* deletion in XL280 RF
ZhaoLab0208/YZ	GATACTGTACTGGCGCTGT	*SWD3* deletion in XL280 RR
ZhaoLab0209/YZ	TGAGATATGAGGGGAATG	*SWD3* deletion in XL280 NRR
ZhaoLab0202/YZ	AAAGGCAACAGAAAGCACGG GTTTTAGAGCTAGAAATAGCAAGTT	*SWD3* deletion in XL280 gRNA, paired with 0192 (gRNA terminator FR)
ZhaoLab0203/YZ	CCGTGCTTTCTGTTGCCTTT AACAGTATACCCTGCCGGTG	*SWD3* deletion in XL280 gRNA, paired with 0193 (U6 promoter FLF)
ZhaoLab0576/RL	CTCCAGTGCCTCCTCAAT	*SWD3* deletion in XL280 Test F
ZhaoLab0577/RL	CGTCTACGTCCTGTTAGCG	*SWD3* deletion in XL280 Test R
ZhaoLab0243/YZ	ATAAGGCCGGCCGCAATTTCAACAA CTGCC	*SWD3* forward with FseI
ZhaoLab0244/YZ	CCTTAATTAACTATTCTGCGTCCCTC CA	*SWD3* reverse with PacI
ZhaoLab0129/YZ	ATAGGCTGGTGCTGTGAATTAAG	XL280 α
ZhaoLab0130/YZ	TGCAGTCACAGCACCTTCTATAC	XL280 α
ZhaoLab0131/YZ	GTTCATCAGATACAGAGGAGTGG	XL280 **a**
ZhaoLab0132/YZ	CTCAACTCTACTTCACCTCACAC	XL280 **a**
ZhaoLab0044/YZ	TCTGACGCTGCGCCTTTG	*RTF1* deletion in XL280 LF
ZhaoLab0045/YZ	CTGGCCGTCGTTTTACATGGGATGC TGATGAGATTGCT	*RTF1* deletion in XL280 LR
ZhaoLab0046/YZ	GCTTCGGGCACCACTAAC	*RTF1* deletion in XL280 NLF
ZhaoLab0047/YZ	GTCATAGCTGTTTCCTGGGGAGAAG GATGACTACGA	*RTF1* deletion in XL280 RF

ZhaoLab0048/YZ	AAAGCGAACTGTGGACGA	RTF1 deletion in XL280 RR
ZhaoLab0049/YZ	GACAAGAAAAGAAACCGA	RTF1 deletion in XL280 NRR
ZhaoLab0050/YZ	AGCATGGTAGGCCAAAGTACGTTTTA GAGCTAGAAATAGCAAGTT	*RTF1* deletion in XL280 gRNA, paired with 0192 (gRNA terminator FR)
ZhaoLab0051/YZ	GTACTTTGGCCTACCATGCT AACAGTATACCCTGCCGGTG	*RTF1* deletion in XL280 gRNA, paired with 0193 (U6 promoter FLF)
ZhaoLab0275/YZ	AGAGTGCGAGGGTTAGTAGG	*RTF1* deletion in XL280 Test F
ZhaoLab0276/YZ	GACAAGCAAAGCCCGAGT	*RTF1* deletion in XL280 Test R
ZhaoLab0306/YZ	GTGGCGGTGGGCCGGCCTCTGACC TCGAGAACGAGCTTT	*RTF1* forward with FseI for FLAG tag
ZhaoLab0307/YZ	CTGCTACTGTAACCCTTAATTCAGAA ATCTCCCAAATCTAGATCCAGCTG	*RTF1* reverse with PacI for FLAG tag
ZhaoLab0089/YZ	GTCCCGGACTCTTCTTGT	*RAD6* deletion in XL280 LF
ZhaoLab0090/YZ	CTGGCCGTCGTTTTACGATGACGGC GTTCCATAC	*RAD6* deletion in XL280 LR
ZhaoLab0091/YZ	TCAGAAGAATAGGACAAAGGAT	*RAD6* deletion in XL280 NLF
ZhaoLab0092/YZ	GTCATAGCTGTTTCCTGTCCGCTAAT CATCAAGGG	*RAD6* deletion in XL280 RF
ZhaoLab0093/YZ	ACCGACTGGTCTCAGCACC	*RAD6* deletion in XL280 RR
ZhaoLab0094/YZ	CTCCGACACTGCTTCAAGAG	*RAD6* deletion in XL280 NRR
ZhaoLab0095/YZ	ATGCTCACCTAGCCGGGTTGGTTTTA GAGCTAGAAATAGCAAGTT	*RAD6* deletion in XL280 gRNA, paired with 0192 (gRNA terminator FR)
ZhaoLab0096/YZ	CAACCCGGCTAGGTGAGCATAACAG TATACCCTGCCGGTG	*RAD6* deletion in XL280 gRNA, paired with 0193 (U6 promoter FLF)
ZhaoLab0281/YZ	ACTGTTCGCTTTATCTCCA	*RAD6* deletion in XL280 Test F
ZhaoLab0282/YZ	ATCTCATCTGCGTTGTCC	*RAD6* deletion in XL280 Test R
ZhaoLab0123/XC	ATAACTCGAGATAATAGGAGTCACCC AGTG	*RAD6* forward with XhoI for GFP tag
ZhaoLab0124/XC	ATAAGCGGCCGCTGATGAGCTCCCT TCGTC	*RAD6* reverse with NotI for GFP tag
ZhaoLab0234/YZ	GTGGCGGTGGGCCGGCCAACGCAG ACCTCAAAAGGGTT	*BRE1* forward with FseI for FLAG tag
ZhaoLab0235/YZ	CTGCTACTGTAACCCTTAATTTATTG CCAATACAGTGTTTGTATATCTTCCT TCG	*BRE1* reverse with PacI for FLAG tag

ZhaoLab0792/RL	CTCTGGTTGGCACGGTG	real time primer for testing JEC21 DNase effect
ZhaoLab0793/RL	CGTCGGTCAATCTTCTCG	real time primer for testing JEC21 DNase effect
ZhaoLab0794/RL	CGTCACCACTGAAGTCAAGT	*TEF1* real time primer
ZhaoLab0795/RL	AGAAGCAGCCTCCATAGG	*TEF1* real time primer
ZhaoLab0593/YJ	AATGGTGGCACGAACGATCT	*CFL1* real time primer
ZhaoLab0594/YJ	GTTGTCGCAATCGGGTTCAG	*CFL1* real time primer
ZhaoLab0595/YJ	GTGATGACGACAAGGAGGCTGTT	*FAD1* real time primer
ZhaoLab0596/YJ	GAGACGCCAGGGATGTTGATGAA	*FAD1* real time primer
ZhaoLab0605/YJ	GCCATCTTACCCCTACCATCTAC	*ZNF2* real time primer
ZhaoLab0606/YJ	TGGACATAGGAACGCTGACAAT	*ZNF2* real time primer
ZhaoLab0601/YJ	TAGCGGAGCGGACTGGAAAGA	*STE3* real time primer
ZhaoLab0602/YJ	CTCGACCGAGACGGCAATCATTA	*STE3* real time primer
ZhaoLab0603/YJ	GCGAATCCACCACCGAATCAATC	*STE6* real time primer
ZhaoLab0604/YJ	CGACGACTGCAACGCACTCT	*STE6* real time primer
ZhaoLab0607/YJ	ATCTTCACCACCTTCACTTCT	*MFalpha2* real time primer
ZhaoLab0608/YJ	CTAGGCGATGACACAAAGG	*MFalpha2* real time primer

**Antibody**	**Item number**	**Dilution**	**Brand**
TriMethyl-Histone H3-K4 Rabbit pAb	A2357	1:2000	ABclonal
DiMethyl-Histone H3-K4 Rabbit pAb	A2356	1:2000	ABclonal
MonoMethyl-Histone H3-K4 Rabbit pAb	A2355	1：2000	ABclonal
Histone H3 Rabbit pAb	A2348	1：5000	ImmunoWay
Ubiquityl-Histone H2B-K120 Rabbit mAb	5546T	1:1000	Cell signaling
Histone H2B Rabbit pAb	A18305	1:2000	ABclonal
Flag-Tag Mouse mAb	AB0008	1:2000	Abways
Goat Anti-Mouse	RS0001	1:20000	ImmunoWay

Goat Anti-Rabbit	RS0002	1:20000	ImmunoWay
