# Supplementary material for: The COMPASS Complex Regulates Fungal Development and Virulence through Histone Crosstalk in the Fungal Pathogen Cryptococcus neoformans
Source: J Fungi (Basel). 2023 Jun 14;9(6):672. doi: 10.3390/jof9060672 (PMC10301970; doi:10.3390/jof9060672)
Supplement: Supplementary file 1 [file jof-09-00672-s001.zip › Supplementary Table S2.pdf]

Supplementary Table S2. Potential Sdc1 subunit in *C. deneoformans* and *C. neoformans*.

Gene ID	Background	Mass/kDa	Function	Localization
CNA06650	XL280	25.41	expressed protein	cytosol
CNA01460	XL280	9.02	expressed protein	mitochondrion
CNG01680	XL280	17.6	hypothetical protein	mitochondrion
CNA06060	XL280	24.64	hypothetical protein	mitochondrion
CNN01130	XL280	26.95	hypothetical protein	peroxisome
CNI02100	XL280	29.81	expressed protein	nucleus
CND06330	XL280	39.6	expressed protein	nucleus
CNB05630	XL280	19.25	hypothetical protein	mitochondrion
CND02180	XL280	31.02	hypothetical protein	mitochondrion
CNC01350	XL280	19.03	expressed protein	Plasma membrane
CNC02620	XL280	21.67	expressed protein	Plasma membrane
CNI03780	XL280	17.1	expressed protein	nucleus
CNE02660	XL280	23.76	conserved hypothetical protein	nucleus
CNI03230	XL280	36.36	conserved hypothetical protein	nucleus
CNC02870	XL280	36.08	expressed protein	nucleus
CNAG_02006	H99	39.71	protein N-terminal asparagine amidohydrolase, putative	mitochondrion
CNAG_03396	H99	52.14	NAD+ diphosphatase, putative	peroxisome
CNAG_03360	H99	49.06	hypothetical protein	nucleus

CNAG_03528	H99	115.17	vesicle-mediated transport-related protein, putative	mitochondrion
CNAG_03886	H99	84.37	expressed protein	cytosol
CNAG_00718	H99	89.32	chromatin assembly complex protein, putative	cytosol
CNAG_04313	H99	43.23	NADPH dehydrogenase 2, putative	cytosol
CNAG_05613	H99	192.94	cellular morphogenesis-related protein, putative	Plasma membrane
CNAG_06548	H99	112.42	endoplasmic reticulum protein, putative	Plasma membrane
CNAG_06356	H99	52.14	mitochondrion protein, putative	Plasma membrane
CNAG_07667	H99	84.04	serine/threonine protein kinase FSK, putative	nucleus
CNAG_03592	H99	61.71	phosphomethylpyrimidine kinase, putative	cytosol
CNAG_05386	H99	56.21	glutamate 5-kinase, putative	mitochondrion
CNAG_07464	H99	82.94	Mbp1 and Swi4-like APSES protein 1	cytosol
CNAG_02366	H99	55.11	4-aminobutyrate aminotransfe	mitochondrion

			rase, putative	
CNAG_02415	H99	62.48	conserved hypothetical protein	mitochondrion
CNAG_02088	H99	52.14	expressed protein	mitochondrion
CNAG_04639	H99	108.9	hypothetical protein	nucleus
CNAG_05768	H99	54.23	regulation of carbohydrate metabolism-related protein, putative	nucleus
CNAG_01824	H99	170.39	transmembr ane protein, putative	Plasma membrane
CNAG_02240	H99	82.28	cytoplasm protein, putative	nucleus
CNAG_05195	H99	125.62	hypothetical protein	nucleus
CNAG_00556	H99	50.49	casein kinase I, putative	nucleus
CNAG_00020	H99	109.56	expressed protein	cytosol
CNAG_00876	H99	64.79	ferric-chelate reductase, putative	Plasma membrane
CNAG_00690	H99	45.21	conserved hypothetical protein	cytosol
