# Supplementary figures and images for: The COMPASS Complex Regulates Fungal Development and Virulence through Histone Crosstalk in the Fungal Pathogen Cryptococcus neoformans
Source: J Fungi (Basel). 2023 Jun 14;9(6):672. doi: 10.3390/jof9060672 (PMC10301970; doi:10.3390/jof9060672)

**A**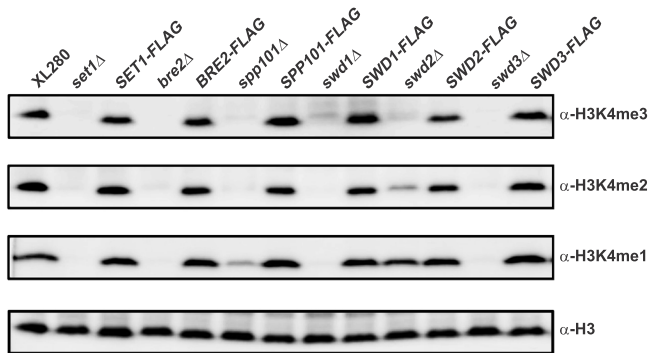**B**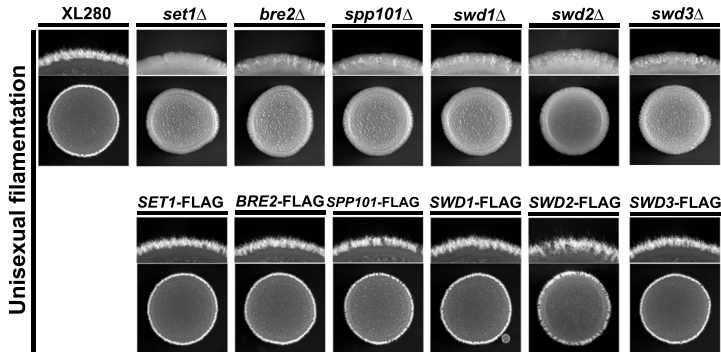

Supplement: Supplementary file 1 [file jof-09-00672-s001.zip › Supplementary Figure S1.pdf]

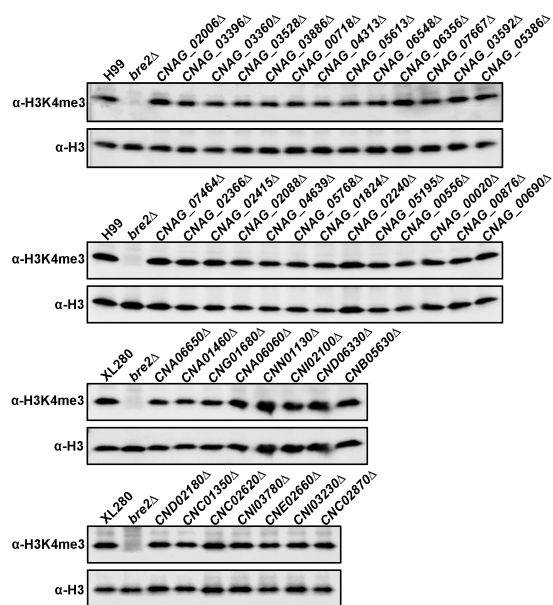

Supplement: Supplementary file 1 [file jof-09-00672-s001.zip › Supplementary Figure S2.pdf]

**A**

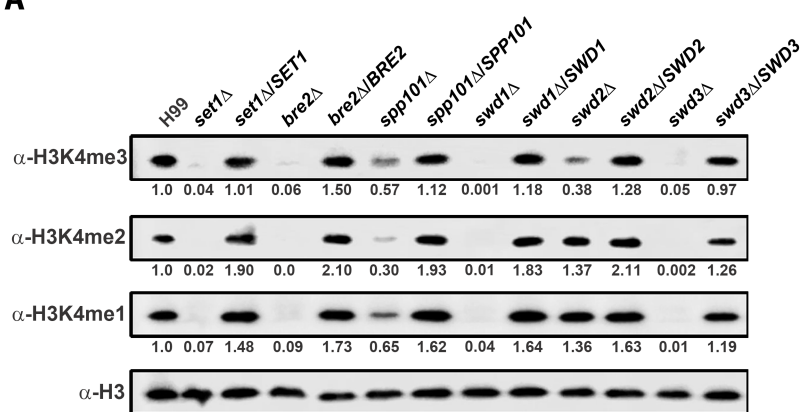

**B**

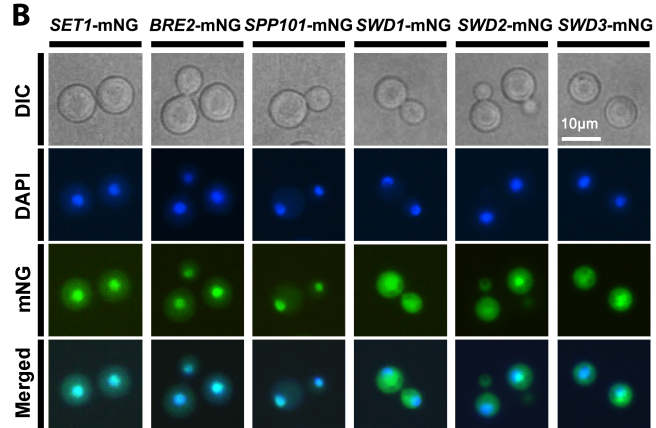

Supplement: Supplementary file 1 [file jof-09-00672-s001.zip › Supplementary Figure S3.pdf]

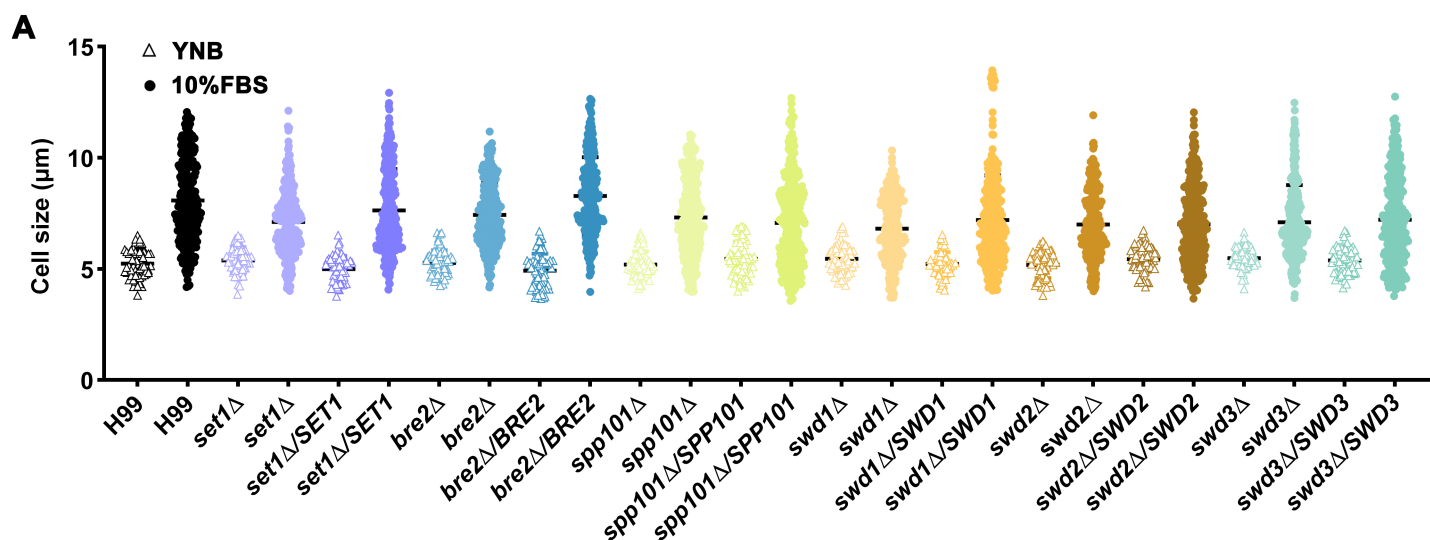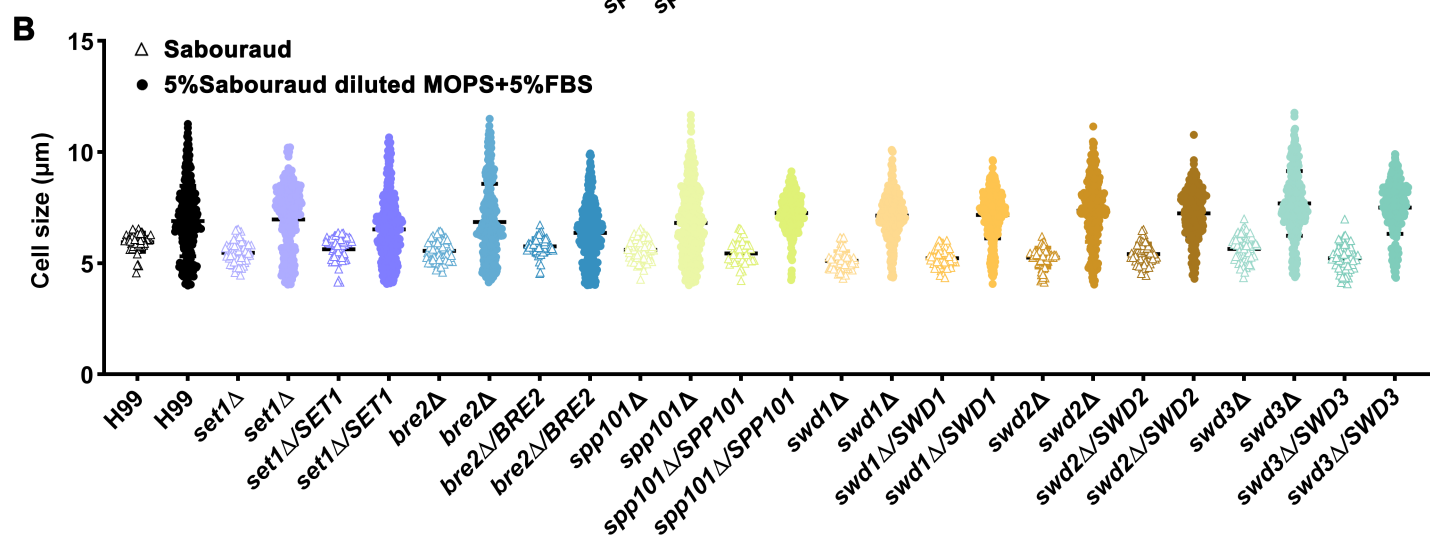

Supplement: Supplementary file 1 [file jof-09-00672-s001.zip › Supplementary Figure S4.pdf]

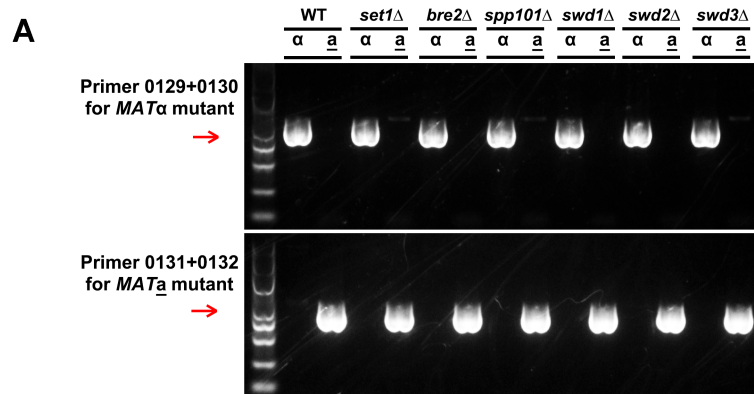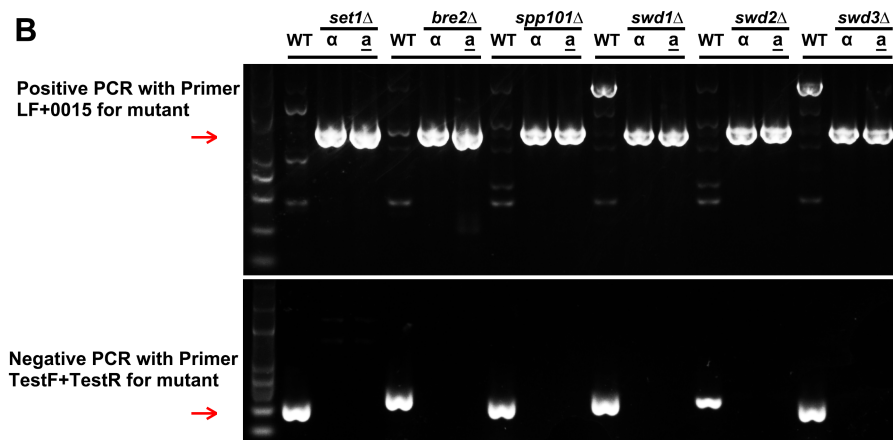

Supplement: Supplementary file 1 [file jof-09-00672-s001.zip › Supplementary Figure S5.pdf]
